# Bearing witness: Medical education and reflecting on the Holocaust then and now

**DOI:** 10.12688/mep.20451.1

**Published:** 2024-10-01

**Authors:** Amanda M. Caleb, Michelle Schmude

**Affiliations:** 1 Department of Medical Education, Geisinger Commonwealth School of Medicine, Scranton, PA, 18509, USA

**Keywords:** Holocaust; moral courage; professional identity formation; social accountability in medicine; reflection

## Abstract

**Background:**

Despite advocacy from the Association of American Medical Colleges (AAMC) and The *Lancet* Commission on medicine, Nazism, and the Holocaust, Holocaust education is lacking in medical education. To address this gap, students at Geisinger Commonwealth School of Medicine (GCSOM) viewed an Association of American Medical College (AAMC) webinar about medicine during the Holocaust as part of the required curriculum for first year medical students introduced in 2022.

**Methods:**

As part of their doctoring course, Physician and Patient Centered Care (PPCC), students viewed the AAMC webinar “The legacy of the role of medicine during the Holocaust and its contemporary relevance” and participated in two structured reflections: a written reflection on how webinar topics inform students' professional development and a verbal reflection on learning from the Holocaust to develop a sense of moral courage, advocacy, and activism in medicine. Researchers conducted qualitative analysis of written reflections and analyzed session surveys to determine key themes and impact of the session.

**Results:**

Of the 108 enrolled in PPCC, 59 (54.6%) completed a post session Likert scale survey assessing the impact of the webinar on their personal and professional development. As an average, respondents moderately agreed that the webinar impacted their personal and professional development, with 91% slightly, moderately, or strongly agreeing. Additionally, thematic analysis of required written reflections indicated a majority of students (62.5%) identified the need for additional medical humanities education about the Holocaust and its relevance to medicine.

**Conclusion:**

Holocaust education encourages medical students to bear witness to past medical atrocities and critically assess the profession and their personal–professional growth. Continued structured integration of the Holocaust in medical education supports critical self-reflection and the development of morally courageous physicians who endorse and practice social accountability in medicine.

## Introduction

In an LCME survey from 2013, 16% of US and Canadian medical schools reported having required curricular components on the Holocaust ^1^. While that number may have increased in the last 10 years, Holocaust education in medical schools is still not required nor is it standardized, despite its vital importance to understanding bioethics and professionalism in medicine ^2– 4^. Engaged learning about the Holocaust cultivates the necessary moral courage to challenge contemporary problematic ideologies and unethical practices ^4, 5^. The Association of American Medical Colleges (AAMC) has supported efforts to increase Holocaust education in medical schools as part of their launch of The Fundamental Role of Arts and Humanities in Medical Education (FRAHME) initiative. While the AAMC acknowledges the challenges of integrating arts and humanities into medical education in a science-based curriculum, including the issue of time and space within a crowded curriculum ^6^, they have also stressed that the humanities “help learners become better observers and better interpreters to build empathy and to better understand the social histories of their patients ^5^.” Moreover, the 2023 *Lancet* Commission on medicine, Nazism, and the Holocaust emphasized the importance of “history-informed professional identity formation” that is essential to addressing contemporary ethical issues and honoring human dignity ^4^.

In 2021, Geisinger Commonwealth School of Medicine (GCSOM) launched its Total Health Curriculum for the MD program, which includes the integration of six themes: community immersion, health systems citizenship, personal and professional development, population health, primary care, and social justice and health equity. These themes augment basic and clinical science education and help foster the development of socially accountable leaders in the health system and the community, an approach that engages with the community to prioritize health needs and to hold medicine accountable for fulfilling its social contract ^6, 7^. Fundamental to this vision is a commitment to health equity, including learning about structural and historical factors that have contributed to contemporary inequities, as well as cultivating a sense of advocacy.

In support of its newly launched curriculum, we integrated viewing and reflecting upon the AAMC-sponsored lecture “The legacy of the role of medicine during the Holocaust and its contemporary relevance” into our first-year doctoring course, Physician and Patient Centered Care, in 2022. The session focused on the themes of social justice, health equity, and personal and professional development and was framed through a humanities-based approach grounded in history and ethics. Our use of the widely available AAMC webinar and the accompanying reflection prompts adds standardization and consistency in Holocaust content delivery and is supported by our standardized reflection curriculum.

## Methods

All first-year medical students were required to view the webinar and respond to reflection prompts published by the AAMC in conjunction with the webinar ^8^. In preparation for class, students provided a written response to the prompt: Reflect on your experience, including your emotional experience, of viewing the seminar, “The legacy of the role of medicine during the Holocaust and its contemporary relevance”: how does the content relate to your being and becoming a physician? ^8^. During class time, students shared these reflections and participated in small-group reflection on a second prompt: Reflecting on the history of perpetrators as well as those who exhibited moral courage/resistors, how might you think about the moral courage you bring to inevitable moral challenges and complexities in the practice of medicine? How might the history of medicine during the Holocaust inform your advocacy and activism as a health care practitioner and/or as a global citizen? ^8^. These prompts encouraged students to make connections between the historical past, their current medical education, and their future responsibilities as physicians.

Reflection is a cornerstone activity for integrating humanities into medical education and allows students to learn from historical events, personal experiences, and clinical encounters alike, fostering the development of new perspectives on that which seems familiar and routine ^9^. Both written and oral reflections can help achieve this goal: written reflection encourages organization of thoughts, revision of ideas, and deep personal connections in a safe space; its permeance permits students to return to their reflections and continue their personal and professional development. Oral reflection creates a safe space in which students can learn from each other and develop a community of trust, responsibility, and accountability. Learning about and reflecting on historical events and medicine, particularly in medicine’s failure to uphold its ethical principles, cultivates a moral obligation to address injustices in medicine ^9^.

Students were also asked to complete a 12 question post-session survey: 3 questions were designed in-house and intended to capture students’ perception of how the AAMC webinar and their accompanying reflections impacted their personal and professional development to patient care ^10^. These questions were validated by the research team using face validity. Questions were modified and 3 were determined to be appropriate for the survey. The other 9 questions were from the validated Moral Courage Scale for Physicians (MCSP) ^11^.

Researchers conducted a mixed method cross-sectional study of anonymized written reflections and volunteer surveys from first-year medical students (analysis of oral reflections was not included in this study). The research team was composed of two faculty members, both of which have experiences with qualitative and mixed methods research and teaching in the Total Health Curriculum at GCSOM. Reflections were collected from Canvas, the Learning Management System and assigned random numbers; survey results were collected via Qualtrics. The research team conducted a thematic analysis of the 108 students’ reflections using a code book they created for the analysis and employing the Delphi method to achieve inter-rater reliability and standard criteria for trusthworthiness ^12^.

Of a possible 108, 59 first-year medical students responded to the voluntary survey (54.6%). The survey employed a 7-point Likert scale with options ranging from strongly disagree (1) to strongly agree (7). Likert scale data were analyzed to determine mode, median, and frequency of positive responses, grouping results of 5 (slightly agree), 6 (moderately agree), and 7 (strongly agree) together.

This study met criteria for exemption by the Geisinger Institutional Review Board (#2019-0293) as defined by the United States Department of Health and Human Services Regulations for the Protection of Human Subjects (45 CFR 46.104 category 1). This includes exemption from documentation of informed consent.

## Results

 Analysis of survey data indicated a positive attitude toward the AAMC webinar and reflection, as well as a dedication to personal and professional values and moral courage. The first survey question, “The AAMC webinar and reflection activities contributed to my personal development,” had a median and mode of 6 (moderately agree); >93% of respondents indicated a positive response (range 1–7). In addition, students were asked if “The AAMC webinar and reflection activities contributed to my professional development.” The median was 6 and the mode was 7 with 91% of students responding positively (range 1–7). Then students were asked if “the AAMC webinar and reflection activities will contribute to my approach to patient care.” The median was 6 and the mode was 7, with 86% responding positively (range 1–7). Because 86%-93% responded positively to each question, we believe the webinar and the reflection experiences enhanced students' personal and professional development and will contribute to their future approach to patient care.

 As part of the MCSP, students were asked, “When faced with ethical dilemmas in patient care, I consider how both my professional values and my personal values apply to the situation before making decisions.” The median and mode were 7, with 97% responding positively (range 4–7). Similarly, students were asked, “I am determined to do the right thing for my patients.” The median and mode score were 7, with 98% responding positively (range 4–7). The responses indicate a commitment to personal and professional development and moral courage that align with the content of the AAMC webinar and associated reflections.

Faculty noted the following themes in 70 of the 108 reflections: the need for additional medical humanities education and training to provide a foundation for continued life-long learning and the need for additional conversations about the Holocaust and its relevance to medicine. One student noted, “At the beginning of the seminar, the AAMC's president emphasized the importance of integrating art and humanities into the science of medicine. I believe this is an essential part of humanizing medicine. Specifically, it is bringing medicine closer to its core of treating the person before the disease.” Another student stated, “It is our duty to honor the legacy of those who lost their lives during the Holocaust. We can start to accomplish such a monumental task by learning about their stories. [L]earning about history will help us write a better, more just future.” Such testimony from students demonstrates the powerful impact of integrating a humanities-based approach to personal and professional development that encourages critical engagement with social justice issues past and present ^4^.

## Discussion

The AAMC webinar “The legacy of medicine during the Holocaust and its contemporary relevance” and the accompanying reflection sessions provided the opportunity to bear witness to medical genocide and to engage in self-reflection about medical ethics and moral courage in medicine. Such deep reflection led students to think more critically about the profession: “as a future physician, I understand that I will have blood on my hand, and by entering this career path, I am also entering the legacy that medicine has had--both the good and the bad.” It also led to critical self-reflection: “It made me question whether or not I was being sufficiently critical of my actions and my education. Do I just accept what I am taught, or do I reflect and question?” This type of reflection is the aim of incorporating historical knowledge within the context of contemporary issues that is essential to addressing health inequities and social injustices through structured reflection.

The pilot integration of the AAMC webinar and associated reflections demonstrated that students found personal and professional value in the session that supports their commitment to being morally courageous physicians. Although results are limited by the lack of pre-session evaluation, such as previous knowledge of the Holocaust, the qualitative analysis supports the value of Holocaust and humanities-based education in professional identify formation and commitment to social justice and health equity among GCSOM students.

GCSOM’s commitment to social justice and health equity, personal and professional development, and reflection linked to the medical humanities support efforts to foster “history-informed professional identify formation ^4^.” The integration of the required AAMC webinar and associated reflections into the preclinical curriculum serves as an anchor for many other existing topics within the curriculum, including medical ethics, social determinants of health, structural racism, and immigration health, as well as supporting professional identity formation of morally courageous physicians. As such, this session has continued to be a required component of the curriculum, with further development of workshop questions and reflections associated with the webinar content.

Our reflection curriculum continues to be refined to support our goal of developing socially accountable leaders in healthcare through “history-informed professional identity formation” ^4^ and encouraging students to not just bear witness but also to speak truth to power. This includes the integration of narrative medicine workshops that focus on issues of social justice in medicine and the deployment of a fourth-year elective on the medical legacy of eugenics and scientific racism. Research suggests that humanities-based medical education is strongest when it is informed by a diversity of voices (including patients and humanities scholars) and when students are provided the space to engage with and reflect on medicine and the medical profession ^6, 9^. Integration of Holocaust education into the medical curriculum allows for this essential reflection to occur through both a painful acknowledgement of the profession’s past and a commitment to “never again” for all populations made vulnerable.

The AAMC affirms that “the arts and the humanities are the connective tissues we need to heal, and we need to thrive ^5^.” Building on resources such as the AAMC seminar “The legacy of medicine during the Holocaust and its contemporary relevance” and our accompanying reflection sessions, we can create this needed space for healing and thriving for future generations of socially accountable and morally courageous physicians.

## Ethical approval

This study was approved by the institutional review board at Geisinger Commonwealth School of Medicine on May 2, 2019 and modified to include additional key personnel on November 4, 2021. Researchers did not seek written informed consent from students whose data were included from this curriculum assignment as all data are reported in aggregate, and individual identifiers are not included. This study met criteria for exemption by the Geisinger Institutional Review Board (#2019-0293) as defined by the United States Department of Health and Human Services Regulations for the Protection of Human Subjects (45 CFR 46.104 category 1). This includes exemption from documentation of informed consent.

## Data Availability

Figshare: Medical student reflections on AAMC webinar “The legacy of the role of medicine during the Holocaust and its contemporary relevance.” https://doi.org/10.6084/m9.figshare.26530708.v1 ^13^ Figshare: Survey of medical students in response to the AAMC webinar “Legacy of Medicine during the Holocaust and its Contemporary Relevance” and Moral Courage Scale for Physicians. https://doi.org/10.6084/m9.figshare.26936989 ^14^ The projects contain the following underlying data: - Holocaust reflections AAMC webinar - Survey answers Data are available under the terms of the Creative Commons Attribution 4.0 International license (CC-BY 4.0). Figshare: Survey tool for AAMC webinar “The legacy of the role of medicine during the Holocaust and its contemporary relevance” and accompanying reflection activities. https://doi.org/10.6084/m9.figshare.25852627.v2 ^10^ This project contains the following extended data: - Survey form Data are available under the terms of the Creative Commons Attribution 4.0 International license (CC-BY 4.0).
