# Supplementary material for: Bearing witness: Medical education and reflecting on the Holocaust then and now
Source: MedEdPublish (2016). 2024 Oct 1;14:205. [Version 1] doi: 10.12688/mep.20451.1 (PMC11484539; doi:10.12688/mep.20451.1)
Supplement: Peer review history

# Reviewer report: Reviewer response for version 1

**DOI:** 10.21956/mep.21886.r39259

**Published:** 2024-11-07

**Authors:** Kathrin Eveline Plank

**Affiliations:** 1 Universitat Passau, Passau, Bavaria, Germany

The brief report addresses a clearly identified gap in medical education and focuses promisingly on the importance of professional reflection. The central research question and the specific connection between the survey and the outlined gap in medical education should be further elaborated.

 The paper outlines an important contribution both in the field of professional research and to the curricular development of medical programs. It clearly highlights the potential and significance of a coherent integration focused on reflecting on professional roles and fields of practice, particularly through engagement with the Holocaust, thereby emphasizing the urgency of addressing the gap identified at the beginning.

 The qualitative methodological approach and corresponding content analysis yield the most relevant results. Here, the underlying guiding questions are justified and made accessible. To assess the quality of the survey, additional information about the survey tool, among other aspects, would be necessary.

 Current and available references are considered. In a further development of this insightful study, it would be important to include literature on professional reflection as well as on the methodological foundations.

Have any limitations of the research been acknowledged?

No

Is the study design appropriate and does the work have academic merit?

Yes

Is the work clearly and accurately presented and does it cite the current literature?

Yes

If applicable, is the statistical analysis and its interpretation appropriate?

Not applicable

Are all the source data underlying the results available to ensure full reproducibility?

Yes

Are the conclusions drawn adequately supported by the results?

Yes

Are sufficient details of methods and analysis provided to allow replication by others?

Partly

Reviewer Expertise:

Education and Space (Focus: Spatial Educational Disadvantage)                                                                Human Rights Education in Teacher Education (Focus: Holocaust Education for Young Learners)               Democratic-Inclusive School Development (Focus: Pedagogical Architecture)                                             Teacher Professionalization in an International Context (Focus: Professional Reflection)

I confirm that I have read this submission and believe that I have an appropriate level of expertise to confirm that it is of an acceptable scientific standard.

# Reviewer report: Reviewer response for version 1

**DOI:** 10.21956/mep.21886.r39266

**Published:** 2024-10-18

**Authors:** Esteban Gonzalez-Lopez

**Affiliations:** 1 Autonomous University of Madrid, Madrid, Spain

I think this paper excellent and is an important addition to the literature on Medicine, Nazism ant the Holocaust. I really like the method used by the authors with asking for reflection and for the future use of the lessons learned for the present day medicine.

 In my experience, medicine and nursing students feel shocked after be informed of the atrocities committed by health-care providers during Nazism and the Holocaust. Many doctors and nurses were fervent supporters of Nazism and executors of the Nazi Racial Hygiene.

 The approach used by the authors is also very interesting. For future iterations of the webinar or seminar I would recommend the authors to include the same survey before starting the seminar in order to compare the opinions of the students before and after (as a suggestion see at https://www.ima.org.il/MedicineIMAJ/Article.aspx?aId=4487 )

 The role played by doctors and nurses in Nazism and The Holocaust must be included in the curricula as a required content because probably is the darkest period in the History of Medicine and there are many lessons to learn from it. The curricula of Medicine and Nursing are completely overcrowded but we must use some other alternatives such us including the topic in History of Medicine, Bioethics, Professionalism etc. Another way for implementing the topic would be to design short seminars or webinars as it was used for the authors.

 I would like to made another suggestion, the term “genocide” has to changed into atrocities, in order to fulfil the criteria (see at https://encyclopedia.ushmm.org/content/en/article/what-is-genocide ) given by Rafael Lempkin

Is the study design appropriate and does the work have academic merit?

Yes

Is the work clearly and accurately presented and does it cite the current literature?

Yes

If applicable, is the statistical analysis and its interpretation appropriate?

Partly

Are all the source data underlying the results available to ensure full reproducibility?

Yes

Are the conclusions drawn adequately supported by the results?

Yes

Are sufficient details of methods and analysis provided to allow replication by others?

Yes

Reviewer Expertise:

Professor of the course, The Holocaust, A Reflection from Medicine. Universidad Autonoma de Madrid, Spain.

I confirm that I have read this submission and believe that I have an appropriate level of expertise to confirm that it is of an acceptable scientific standard.

# Reviewer report: Reviewer response for version 1

**DOI:** 10.21956/mep.21886.r39170

**Published:** 2024-10-18

**Authors:** Stephanie Manca

**Affiliations:** 1 Italian National Research Council, Rome, Italy

The report presents the findings of a study investigating the experiences of a cohort engaged in a medical course on the subject of the Holocaust. The study is well described and the methodology is clearly presented in a manner that allows for replication. It is worth noting that the study is not without some shortcomings. In the Results and Discussion sections, the two authors have combined the presentation of results with their interpretations. It would be preferable to present the results in the Results section and to interpret them in the Discussion in light of previous studies. Furthermore, it would be beneficial to place greater emphasis on the value of the study and its limitations, as well as on the implications for practice and future research.

Have any limitations of the research been acknowledged?

No

Is the study design appropriate and does the work have academic merit?

Yes

Is the work clearly and accurately presented and does it cite the current literature?

Yes

If applicable, is the statistical analysis and its interpretation appropriate?

Partly

Are all the source data underlying the results available to ensure full reproducibility?

Partly

Are the conclusions drawn adequately supported by the results?

Yes

Are sufficient details of methods and analysis provided to allow replication by others?

Yes

Reviewer Expertise:

Holocaust education

I confirm that I have read this submission and believe that I have an appropriate level of expertise to confirm that it is of an acceptable scientific standard, however I have significant reservations, as outlined above.

# Reviewer report: Reviewer response for version 1

**DOI:** 10.21956/mep.21886.r39260

**Published:** 2024-10-16

**Authors:** Melanie Carina Schmoll

**Affiliations:** 1 Bar Ilan University, The Finkler Institute of Holocaust Research, Ramat Gan, Israel

This paper addresses an important topic. Holocaust education is more than just teaching history in schools, but should - as here - also find its way into adult education. It is good to see that Holocaust education is integrated into degree courses, especially  regarding future physicians.

 This study impressively shows how historical and humanities topics and questions are linked to medical issues. Questions about values and attitudes against the background of the Holocaust have not only found their way into medical studies. Still, they are also scientifically monitored and evaluated should be particularly emphasized.

 With regard to the evaluation method used, the division into written and oral statements is a clever idea. Particularly in view of the fact that research on Holocaust education among pupils (who are not particularly far removed in age from students in the first semester) has shown that in quite a few cases pupils give *socially desirable answers *with regard to the pressing questions on the subject of the Holocaust. A division into both forms of feedback may counteract a bias due to *socially desirable answers*.

 It should be noted that it would have been helpful to ask questions about knowledge about the Holocaust before the seminar. No student comes to a seminar like this as a *blank slate*.

 Holocaust education can be used to sensitize people, fostering their sensitivity to values such as the preciousness of human life and life in general. Given that, Holocaust education can be seen as a basis for knowledge and *peace education* in the sense of Galtung – and not only in the sense of global citizenship as mentioned in the text.

 The study would be even more meaningful if least the possibility of *socially desirable answers* was recognized and clearly stated. Especially since the webinar was compulsory.

 Two other small points could be mentioned: The title is a little confusing (then and now?).

 The term genocide has a precise legal definition and does not apply here. The term was precisely defined by the UN in 1948. For the term genocide to be applied to the mass killing of a group of people, all five acts defined by the Convention must have been committed. Accordingly, genocide can be said to have been committed if it involves, the killing of members of the group, if these acts cause serious bodily or mental harm to members of the group, if these acts intentionally impose living conditions on the group concerned which are likely to bring about their physical destruction in whole or in part. In addition, measures aimed at preventing births within the group must have been imposed and involve the forcible transfer of children from the group to another group.

Have any limitations of the research been acknowledged?

Yes

Is the study design appropriate and does the work have academic merit?

Yes

Is the work clearly and accurately presented and does it cite the current literature?

Yes

If applicable, is the statistical analysis and its interpretation appropriate?

I cannot comment. A qualified statistician is required.

Are all the source data underlying the results available to ensure full reproducibility?

Yes

Are the conclusions drawn adequately supported by the results?

Yes

Are sufficient details of methods and analysis provided to allow replication by others?

Yes

Reviewer Expertise:

Dr. Schmoll is a historian and Holocaust Education expert. Her work focuses on National Socialism and the Holocaust, Holocaust Education, education policy and tools and aspects of remembrance and memory.

I confirm that I have read this submission and believe that I have an appropriate level of expertise to confirm that it is of an acceptable scientific standard.

# Reviewer report: Reviewer response for version 1

**DOI:** 10.21956/mep.21886.r39263

**Published:** 2024-10-15

**Authors:** Tessa Chelouche

**Affiliations:** 1 Rappaport Faculty of Medicine, Technion - Israel Institute of Technology, Haifa, Israel

This is an important paper that adds to the developing body of research on the importance of the inclusion of the history of medicine, Nazism and the Holocaust into the medical curriculum. 

 It is very pleasing and encouraging to note that GCSOM is committed to this difficult ,but essential, task of including this specific content of medical humanities into the curriculum and that it is recognized that this is important for the professional identity of future medical professionals .

  It is also good to see that oral and written reflections were used as a pedagogical tool, which are essential for learning this history.

 It would have been of added value had pre-session evaluations been obtained.

 Line 2 of the discussion: the term ' **genocide**' needs to be changed to something like  'atrocities'. Genocide has a very specific definition and the medical atrocities permitted in Nazi Germany do not entirely line up with this definition.

Have any limitations of the research been acknowledged?

Yes

Is the study design appropriate and does the work have academic merit?

Yes

Is the work clearly and accurately presented and does it cite the current literature?

Yes

If applicable, is the statistical analysis and its interpretation appropriate?

I cannot comment. A qualified statistician is required.

Are all the source data underlying the results available to ensure full reproducibility?

Yes

Are the conclusions drawn adequately supported by the results?

Yes

Are sufficient details of methods and analysis provided to allow replication by others?

Yes

Reviewer Expertise:

The history of medicine, Nazism and the Holocaust and the implications, mainly ethical,  for the present and the future.

I confirm that I have read this submission and believe that I have an appropriate level of expertise to confirm that it is of an acceptable scientific standard.
